# Additional Fertilizer and Nematicide Combinations on Upland Cotton to Manage *Rotylenchulus Reniformis* and *Meloidogyne Incognita* in Alabama

**DOI:** 10.21307/jofnem-2022-003

**Published:** 2022-02-18

**Authors:** Kara L. Gordon, Drew W. Schrimsher, Kathy S. Lawrence

**Affiliations:** 1 Auburn University, 559 Devall Dr. CASIC Building, Auburn, AL 36849.

**Keywords:** Cotton, *Meloidogyne*, Reniform nematode, Root-knot nematode, *Rotylenchulus*

## Abstract

Plant parasitic nematodes are major pests on upland cotton worldwide and in the United States. The reniform nematode, *Rotylenchulus reniformis* and the southern root-knot nematode *Meloidogyne incognita* are some of the most damaging nematodes on cotton in the United States. Current management strategies focus on reducing nematode populations with nematicides. The objective of this research was to integrate additional fertilizer and nematicide combinations into current practices to establish economical nematode management strategies while promoting cotton yield and profit. Microplot and field trials were run to evaluate fertilizer and nematicide combinations applied at the pinhead square (PHS) and first bloom (FB) plant growth stages to reduce nematode population density and promote plant growth and yield. Cost efficiency was evaluated based on profit from lint yields and chemical input costs. Data combined from 2019 and 2020 suggested a nematicide seed treatment (ST) ST + (NH_4_)_2_SO_4_ + Vydate^®^ C-LV + Max-In^®^ Sulfur was the most effective in increasing seed cotton yields in the *R. reniformis* microplot trials. In *R. reniformis* field trials, a nematicide ST + (NH_4_)_2_SO_4_ + Vydate^®^ C-LV at PHS supported the largest lint yield and profit per hectare at $1176. In *M. incognita* field trials, a nematicide ST + 28-0-0-5 + Vydate^®^ C-LV + Max-In^®^ Sulfur at PHS and FB supported the largest lint yields and profit per hectare at $784. These results suggest that combinations utilizing fertilizers and nematicides applied together across the season in addition to current fertility management show potential to promote yield and profit in *R. reniformis* and *M. incognita* infested cotton fields.

Cotton (*Gossypium spp.*) is one of the most important fibers produced worldwide and is a staple in the United States and global economies. An estimated 14 million acres of cotton are harvested in the United States equating to $38 billion dollars each year (Wilkins et al., 2000). Upland cotton (*Gossypium hirsutum* L.) is the most widely grown cotton species in the United States and worldwide, consisting of nearly 90% of total cotton production (Glade et al., 1996; Wakelyn et al., 2006). Cotton is restricted to tropical and subtropical regions because of elevated temperatures and humidity that are ideal for growth (Luttrell et al., 1994). These climate conditions are found in the cotton belt of the Southern United States where most cotton production occurs (Jones and Durand, 1959).

The reniform nematode (*Rotylenchulus reniformis* Linford and Oliveira) and the southern root-knot nematode (*Meloidogyne incognita* Kofoid and White) are the most economically important nematodes on upland cotton (*Gossypium hirsutum*) (Robinson, 2007). Nearly 182 thousand hectares of upland cotton were planted in Alabama in 2020 (USDA, 2021). In the same year, *M. incognita* and *R. reniformis* caused a combined estimated yield loss of approximately 22 thousand hectares, representing a 12% yield loss in Alabama (Lawrence et al., 2021). *Rotylenchulus reniformis* damage include stunted plants due to limited root development that produce a wave-like pattern across the canopy (Lawrence and Lawrence, 2020), reduced size and number of bolls that result in reduced lint yield (Jones et al., 1959). *Meloidogyne incognita* visual aboveground symptoms include stunted plant growth and wilting due to reduced water and nutrient uptake (Davis and May, 2003; Lawrence and Lawrence, 2020). The most identifiable symptoms are the massive galls formed on host root system (Chitwood, 1949).

Common nematode management strategies include crop rotation, nematicides and host resistance (Starr et al., 2007). High rates of fertilizer have been documented to limit nematode induced crop damage (Chawla and Prasad, 1973), reduce plant stress, and promote plant growth (Whitaker et al., 2018). However, there is very little research done on the effect of fertilizer on cotton production systems that are infested with nematodes. Most research is conducted either on the efficacy of nematicides (Lawrence et al., 1990) or the importance of nitrogen fertilizer in cotton (Duncan and Raper, 2019). Integrating nematicides and additional fertilizer applications into a single management regiment has the potential to simultaneously limit nematode induced damage and promote plant growth.

Ideal management strategies utilize a combination of the most effective and cost-efficient practices based on nematode species and levels, financial resources and environmental conditions (Grabau, 2017). Nematicides are commercially available to growers in a variety of applications. Seed treatments and foliar sprays can adequately reduce *R. reniformis* and *M. incognita* populations (Faske and Hurd, 2015; Lawrence and McLean, 2002), and tend to be the most common treatments. A study conducted by Lawrence et al. (2015) found a combination of the nematicide seed treatment Aeris^®^ (thiodicarb) with two foliar applications of Vydate^®^ C-LV (oxamyl) applied after germination and the 2 to 8 leaf stage reduced *R. reniformis* and *M. incognita* populations. Commercially available fertilizers can be applied as a broadcast, dribbled, knifed-in or banded during planting (Oldham, 2017). Fertilizer amendments have shown nematicidal effects while simultaneously promoting yield (Muller and Gooch, 1982). Sidedress applications between first square and first bloom are suggested for growers in the Southeast (Whitaker et al., 2018). A study conducted by Mullins et al. (2003) suggested that fertilizer applied during peak uptake, considered to be during early bloom and peak bloom, has the potential to increase lint yields between 8.4 and 13.7%.

Maximizing profit is the most important factor in any cotton production system. Management strategies for pests and fertility programs play a huge role in the economic viability of the cotton-cropping program. The primary objective of this study was to analyze the impact of adding additional fertilizer and nematicide combinations to cotton grown in *R. reniformis* and *M. incognita* infested fields to optimize cotton yield. The two main objectives of this research were: (i) to evaluate the effects of additional fertilizer and nematicide applications to cotton at pin hear square (PHS) as the plants begin to bloom and again at full bloom (FB) at two critical plant growth stages affecting yield and; (ii) to determine the financial impact of additional fertilizer and nematicide combinations using input costs and revenue.

## Materials and methods

Microplot and field trials were used to evaluate the impact of additional fertilizer and nematicide applications on population levels of *R. reniformis* and *M. incognita* and cotton yield.

### Fertilizers and nematicides

Ammonium sulfate, (NH_4_)_2_SO_4_ and 28-0-0-5 are the standard nitrogen fertilizer blends utilized for cotton production in the southeast and are usually applied as a split application near planting and at pin head square (PHS). Max-In^®^ Sulfur (WinField United, Arden Hills, MN) is an additional sulfur application that maybe added to any spray application applied to cotton usually at PHS. We evaluated splitting fertilizer applications of (NH_4_)_2_SO_4_, or 28-0-0-5 and Max-In^®^ Sulfur across the season at PHS or at PHS and first bloom (FB).

Aeris^®^ (Bayer CropScience, Research Triangle Park, NC) (Imidacloprid and Thiodicarb) was the seed treatment nematicide in 2019 and (Table 1). COPeO^™^ Prime (BASF, Florham Park, NJ) (Fluopyram) was the seed treatment nematicide in 2020. Seed treatments were applied to the DP 1646 B2XF cotton seed before planting using a Gustafson laboratory tabletop seed treater (Pinckard, 1971). Seeds were air dried for 24 hr before planting. The additional nematicide Vydate^®^ C-LV (DuPont, Wilmington, DE) (Oxamyl) was applied as a foliar spray at pinhead square (PHS) and/or first bloom (FB) (Table 1).

**Table 1:** Additional fertilizer and nematicide rates and application method used in Plant Science Research Center, Auburn, AL microplots and Tennessee Valley Research and Extension Center, Belle Mina, AL, and Plant Breeding Unit, Shorter, AL field trials in 2019 and 2020.

**Chemicals^z^**	**Microplot rate**	**Field rate**	**Application**
(NH_4_)_2_SO_4_(21-0-0-24)	0.52 g/m	108 kg/ha	Broadcast
28-0-0-5	0.289 ml/m	89 L/ha	Knifed
Vydate^®^ C-LV (Oxamyl)	0.004 ml/m	0.8 L/ha	Foliar spray
Max-In^®^ Sulfur (0-0-19-13)	0.007 ml/m	1.5 L/ha	Foliar spray
Aeris^®^ (Imidacloprid & Thiodicarb)	0.375 mg ai/seed	0.375 mg ai/seed	Seed treatment
COPeO^™^ Prime (Fluopyram	0.30 mg ai/seed	0.30 mg ai/seed	Seed treatment

**2019**			
**Treatment**	**Fertilizer**	**Nematicides**	**Application**

1	(NH_4_)_2_SO_4_	Untreated	PHS
2	28-0-0-5	Untreated	PHS
3	(NH_4_)_2_SO_4_	Aeris^®^	PHS
4	28-0-0-5	Aeris^®^	PHS
5	(NH_4_)_2_SO_4_	Aeris^®^	PHS + FB
6	28-0-0-5	Aeris^®^	PHS + FB
7	(NH_4_)_2_SO_4_	Aeris^®^ + Vydate^®^ C-LV	PHS
8	28-0-0-5	Aeris^®^ + Vydate^®^ C-LV	PHS
9	(NH_4_)_2_SO_4_ + Max-In^®^ Sulfur	Aeris^®^ + Vydate^®^ C-LV	PHS
10	28-0-0-5 + Max-In^®^ Sulfur	Aeris^®^ + Vydate^®^ C-LV	PHS
11	(NH_4_)_2_SO_4_ + Max-In^®^ Sulfur	Aeris^®^ + Vydate^®^ C-LV	PHS + FB
12	28-0-0-5 + Max-In^®^ Sulfur	Aeris^®^ + Vydate^®^ C-LV	PHS + FB

**2020**			
**Treatment**	**Fertilizer**	**Nematicides**	**Application**

1	(NH_4_)_2_SO_4_	Untreated	PHS
2	28-0-0-5	Untreated	PHS
3	(NH_4_)_2_SO_4_	COPeO^™^	PHS
4	28-0-0-5	COPeO^™^	PHS
5	(NH_4_)_2_SO_4_	COPeO^™^	PHS + FB
6	28-0-0-5	COPeO^™^	PHS + FB
7	(NH_4_)_2_SO_4_	COPeO^™^ + Vydate^®^ C-LV	PHS
8	28-0-0-5	COPeO^™^ + Vydate^®^ C-LV	PHS
9	(NH_4_)_2_SO_4_ + Max-In^®^ Sulfur	COPeO^™^ + Vydate^®^ C-LV	PHS
10	28-0-0-5 + Max-In^®^ Sulfur	COPeO^™^ + Vydate^®^ C-LV	PHS
11	(NH_4_)_2_SO_4_ + Max-In^®^ Sulfur	COPeO^™^ + Vydate^®^ C-LV	PHS + FB
12	28-0-0-5 + Max-In^®^ Sulfur	COPeO^™^ + Vydate^®^ C-LV	PHS + FB

Notes: Applications were at pinhead square (PHS) and/or first bloom (FB) cotton growth stage. Chemicals were applied on the upland cotton cultivar DP 1646 B2XF^z^.

### Treatment combinations

Trials were conducted with 12 fertilizer and nematicide combinations with the idea of continually feeding and protecting the developing cotton plant as it grows and develops cotton bolls. All treatments received the initial at plant base fertilizer of (NH_4_)_2_SO_4_ or 28-0-0-5. Treatment 1 was the control with no seed treatment nematicide on the cotton seeds at planting with an additional PHS application of the granular (NH_4_)_2_SO_4_ fertilizer while treatment 2 is also the control again without a seed treatment nematicide on the cotton seeds with an additional PHS application of the liquid 28-0-0-5 fertilizer (Table 1). Treatments 3 and 4 were the same cotton seeds with a seed treatment nematicide (Aeris in 2019 and COPeO in 2020) with an additional PHS application of the granular (NH_4_)_2_SO_4_ fertilizer for treatment 3 and an additional PHS application of the liquid 28-0-0-5 fertilizer for treatment 4. Treatment s 5 and 6 were the same as 3 and 4 with the addition of a sequential fertilizer application at FB. Treatments 7 and 8 were the same seed treatment nematicide and fertilizers as treatments 3 and 4 with the addition of the nematicide Vydate^®^ C-LV applied at PHS. Treatments 9 and 10 were the same as 7 and 8 with another addition of Max-In^®^ Sulfur added to the Vydate^®^ C-LV applied at PHS. Treatments 11 and 12 were the same as 9 and 10 with the addition of Max-In^®^ Sulfur and Vydate^®^ C-LV applied at PHS and sequentially at FB.

### Microplot evaluations

Microplot trials were conducted in 2019 and 2020 at the Plant Science Research Center (PSRC) in Auburn, AL. Four tests were conducted, one each year for *R. reniformis* and *M. incognita*. Microplots were 26.5 L plastic pots filled with Kalmia loamy sand (24% sand, 49% silt and 28% clay) from the Plant Breeding Unit (PBU) or Decatur silt loam (24% sand, 49% silt and 28% clay) from the Tennessee Valley Research and Extension Center (TVREC) and represent 0.3 m of row in the field. Each microplot was inoculated with 250 cm^3^ of soil containing approximately 50,000 eggs and vermiform life stages of either *R. reniformis* or *M. incognita* and placed in the base of the planting furrow. *Rotylenchulus reniformis* nematode was collected from Tennessee Valley Research and Extension Center near Belle Mina, AL and *M. incognita* from Plant Breeding Unit near Tallassee, AL. Cultures of these nematodes were maintained at the PSRC at Auburn University. The cotton cultivar “Phytogen 340 W3FE” (Corteva Agriscience, Wilmington, DE) was used to maintain *R. reniformis* and *M. incognita* populations were maintained on corn “Mycogen 2H723” (Dow AgroScience, Indianapolis, IN). Microplots received a pre-plant broadcast application of 13-13-13 applied at 0.13kg/m and hand tilled into the soil. Ten cotton seeds, “DP 1646 B2XF” (Bayer CropScience, Research Triangle, NC) pretreated with an insecticide/fungicide seed treatment by Bayer CropScience (metalaxyl, pyraclostrobin, myclobutanil, imidacloprid, fluxapyroxa), were planted 2.5 cm deep into a furrow in each microplot and thinned to five seedlings after germination. Irrigation was administered through a drip irrigation system at 30 ml/min and was adjusted throughout the season to run for 15 to 45 min twice a day.

All tests were arranged in a randomized complete block design (RCBD), with five replications. The first additional application of fertilizers and nematicides were applied at PHS. At PHS, (NH_4_)_2_SO_4_ was applied by hand to the base of the plant. At PHS, 28-0-0-5 was pipetted into a narrow indention created with a hand spade 5 cm beside and 5 cm below the base of treated plants. Max-In^®^ Sulfur and Vydate^®^ C-LV were applied as foliar sprays via a handheld spray bottle. The second application of additional fertilizers was applied at FB. All fertilizers and nematicides were applied identically at PHS and FB. In 2020, microplots received the same management practices as in 2019 with the exception that the nematicide seed treatment Aeris^®^ was replaced with Copeo™ Prime.

### Microplot data collection

Data were collected at PHS and FB. One cotton plant was excavated from each microplot for plant and nematode data at each of the sample data collection times. Plant parameters included plant height (PH), and root fresh weight (RFW) and seed cotton yield. Nematode parameters of *R. reniformis* and *M. incognita* population density included number of eggs per g of root. At plant maturity, cotton was hand harvested for all microplot trials.

### Nematode extraction

To obtain nematode population levels, eggs were extracted from the cotton roots using a modified method of Hussey and Barker (1973). Eggs were collected for *R. reniformis* and *M. incognita* by placing roots in a 0.625% NaOCl solution and shaken for four minutes on a Barnstead Lab Line Max Q 5000 E Class shaker (Conquer Scientific: San Diego, CA). Roots were rinsed with water and scrubbed by hand; eggs were collected on a 25-μm sieve and poured into a 50mL centrifuge tube. The product was further processed by sucrose centrifugation following the modified methodology of Jenkins (1964). Contents were centrifuged at 220 g-forces for 1 min and then rinsed with water; eggs were collected on a 25-μm sieve. Eggs were enumerated via a Nikon TSX 100 inverted microscope at a ×40 magnification.

### Field evaluations

Field trials were conducted at TVREC near Belle Mina, AL, and at PBU near Tallassee, AL. Both research stations maintained plots throughout the growing season with standard herbicide, insecticide, and fertility practices. Fertility practices at both locations included a pre-plant application of 28-0-0-5 at 112 kg/ha in late April followed by a sidedress application of 28-0-0-5 at 34 kg/ha applied in mid-July. TVREC was artificially infested with *R. reniformis* in 2007, the initial population density at planting averaged 5000 vermiform life stages per 100 cm^3^ of soil in 2019 and 2020. The soil type in this field is a Decatur silt loam. PBU is naturally infested with *M. incognita* and the initial population density at planting was 77 J2 per 100 cm3 of soil in a Kalmia loamy sand soil type. The trials were arranged in a RCBD with 5 replications and the entire test was repeated within each year. Both sites were planted using a John Deere MaxEmerge planter (Moline, Illinois) equipped with Almaco cone planters (Nevada, Iowa). Trials were planted with DP 1646 B2XF at a rate of 100 seeds per 7.6 m. Plots at TVREC consisted of 2 rows that were 7.6 m long with 1.01 m row spacing and a 6 m wide alley. Plots at PBU consisted of 2 rows that were 7.6 m long with 0.9 m row spacing and a 6 m wide alley.

Additional fertilizer, nematicides, and seed treatment applications were identical to the microplot trials. Additional fertilizer and nematicide combinations were applied at PHS and FB. Ammonium sulfate was applied by hand to the base of the plant. The 28-0-0-5 was knifed into the soil 5cm beside and 5 cm below the plant with a liquid fertilizer applicator and fertilizer disc. WinField Max-In^®^ Sulfur and Vydate^®^ C-LV (Oxamyl) were applied as a foliar spray at 25 PSI with a Case IH 265 tractor equipped with a 4 boom sprayer at PHS and FB. Entire plots were machine harvested with a Case International Harvester 2555 cotton picker with Harvest Weigh Mobile by System Scales at TVREC and PBU.

### Field data collection

Four representative cotton plants from each plot were randomly dug up from each plot to collect plant and nematode data at PHS and FB. The plant growth parameters included plant stand, plant height, root fresh weight and cotton yield. Nematode population density was collected as in the microplot trials after transport from the field to PSRC. Fifty mature bolls were hand harvested from the first rep of each test. Samples were ginned using a 10-saw table-top gin at the PSRC. The lint and seeds collected from the gin were weighed individually and these data were used to calculate the lint ratio for each treatment. All plots were machine harvested to determine seed cotton yields.

### Data analysis

Data collected from microplot, and field trials were analyzed with SAS 9.4 (SAS Institute, Cary, NC) using the PROC GLIMMIX procedure. LS-means were compared using ANOVA, and Tukey–Kramer multiple pair wise comparison at a significance level of P ≤ 0.10. Dependent variables included plant stand, plant height, root fresh weight, *R. reniformis* and *M. incognita* eggs per gram of root (eggs/g of root), seed cotton yield (kg/ha) and mean ($/kg). Fixed effects comprised of nematicide and fertilizer treatments at PHS and/or FB. Random effects comprised of replication, test repetition and location.

There were no significant interactions between 2019 and 2020 thus the data from both years were combined into a single dataset. Different nematicide seed treatments were used in both years; therefore, we analyzed the effects of a general nematicide seed treatment and not a specific chemical.

### Profit calculation

Revenue was calculated using the most current price from the USDA upland cotton announcement (https://www.fsa.usda.gov/Internet/FSA_EPAS_Reports.pdf) of $1.32/kg in 2019 and $1.54/kg in 2020 and the lint ratio from each treatment. Fertilizer and nematicide input costs were acquired through a local sales agricultural (Stephen Till, personal communications) representative in 2019 and 2020. Input costs were subtracted from revenue to determine profit for individual treatment combinations. Upper, lower, and mean profit for each combination was determined using a confidence interval from an ANOVA test at *P* ≤ 0.10.

## Results

### *Rotylenchulus reniformis* microplot evaluation

In the microplot setting with *R. reniformis*, the additional fertilizer and nematicide applications did not significantly increase plant height or root fresh weight when data collected at PHS and FB were combined (Table 2). *Rotylenchulus reniformis* eggs per gram of root were significantly reduced when data collected at PHS and FB were combined for the nematicide ST + 28-0-0-5 + Vydate^®^ C-LV + Max-In^®^ Sulfur at PHS application compared to the control application of (NH_4_)_2_SO_4_ at PHS with no ST nematicide. A significant increase in seed cotton yield was measured with the maximum input combination of the nematicide ST + (NH_4_)_2_SO_4_ + Vydate^®^ C-LV + Max-In^®^ Sulfur applied at PHS and FB when compared to the combination of the nematicide ST + (NH_4_)_2_SO_4_ applied at PHS (*P* ≤ 0.10).

**Table 2:** Microplot LS means^z^ from 2019 and 2020 of the effect of additional nematicide and fertilizer combinations on DP 1646 B2XF^y^ plant height, cotton root fresh weight at pinhead square (PHS) and first bloom (FB), *Rotylenchulus reniformis* eggs per gram of root at PHS and FB sample data summed, and seed cotton yield at the Plant Science Research Center.

**No**	**Treatments**	**Plant height (cm)**	**Root fresh weight (g) PHS^x^ + FB^w^**	***R. reniformis* eggs/g root-PHS + FB**		**Seed cotton yield (g)**	
1	(NH_4_)_2_SO_4_–PHS	68	12.49	308	a	36	ab
2	28-0-0-5 – PHS	67	12.04	183	ab	33	ab
3	ST^v^ + (NH_4_)_2_SO_4_–PHS	71	15.73	208	ab	28	b
4	ST + 28-0-0-5 – PHS	66	15.12	271	ab	36	ab
5	ST + (NH_4_)_2_SO_4_–PHS + FB	68	20.49	121	ab	45	ab
6	ST + 28-0-0-5 – PHS + FB	67	19.35	252	ab	41	ab
7	ST + (NH_4_)_2_SO_4_ + Vydate^®^ C-LV – PHS	71	12.89	152	ab	41	ab
8	ST + 28-0-0-5 + Vydate^®^ C-LV – PHS	73	16.02	110	ab	47	ab
9	ST + (NH_4_)_2_SO_4_ + Vydate^®^ C-LV + Max-In^®^ Sulfur – PHS	71	15.44	272	ab	47	ab
10	ST + 28-0-0-5 + Vydate^®^ C-LV + Max-In^®^ Sulfur – PHS	73	14.77	70	b	44	ab
11	ST + (NH_4_)_2_SO_4_ + Vydate^®^ C-LV + Max-In^®^ Sulfur – PHS + FB	68	12.93	220	ab	55	a
12	ST + 28-0-0-5 + Vydate^®^ C-LV + Max-In^®^ Sulfur – PHS + FB	73	16.13	147	ab	47	ab

Notes:

z LS-means followed by the same letter are not significantly different at *P* ≤ 0.10 as determined by the Tukey Kramer method.

y All DP 1646 B2XF seeds were pretreated with a company fungicide and insecticide metalaxyl, pyraclostrobin, myclobutanil, imidacloprid, and fluxapyroxad.

x PHS refers to the pinhead square plant growth stage when the first additional combination of fertilizers and nematicides were applied.

w FB refers to the first bloom plant growth stage when the second additional combination of fertilizers and nematicides were applied.

v ST refers to nematicide seed treatment, Aeris^®^ (Thiodicarb) applied in 2019 and COPeO™ Prime (Fluopyram) applied in 2020.

### *Meloidogyne incognita* microplot evaluation

The *M. incognita* infested microplot trials found the additional fertilizer and nematicide combinations did not significantly increase plant height or root fresh weight sampled when data collected PHS and FB were combined (Table 3). *Meloidogyne incognita* eggs per gram of root were lowest in the application of 28-0-0-5 at PHS, and in the combinations of the nematicide ST + 28-0-0-5 + Vydate^®^ C-LV at PHS and a nematicide ST + (NH_4_)_2_SO_4_ + Vydate^®^ C-LV + Max-In^®^ Sulfur at PHS and FB. Seed cotton yields were similar across all fertilizer and nematicide combinations in the microplot tests.

**Table 3:** Microplot LS means^z^ from 2019 and 2020 of the effect of additional nematicide and fertilizer combinations on DP 1646 B2XF^y^ plant height, cotton root fresh weight at pinhead square (PHS) and first bloom (FB) *Meloidogyne incognita* eggs per gram of root at PHS and FB sample data summed, and seed cotton yield at the Plant Science Research Center.

**No**	**Treatments**	**Plant height (cm)**	**Root fresh weight (g) PHS^x^ + FB^w^**	***M. incognita* eggs/g root PHS + FB**	**Seed cotton yield (g)**
1	(NH_4_)_2_SO_4_ – PHS	51	10.81	554 ab	34
2	28-0-0-5 – PHS	52	9.70	130 b	34
3	ST^v^ + (NH_4_)_2_SO_4_ – PHS	55	8.36	405 ab	33
4	ST + 28-0-0-5 – PHS	61	10.35	224 ab	32
5	ST + (NH_4_)_2_SO_4_ – PHS + FB	42	9.41	677 a	33
6	ST + 28-0-0-5 – PHS + FB	45	9.18	309 ab	47
7	ST + (NH_4_)_2_SO_4_ + Vydate^®^ C-LV – PHS	52	9.83	241 ab	24
8	ST + 28-0-0-5 + Vydate^®^ C-LV – PHS	55	9.71	146 b	34
9	ST + (NH_4_)_2_SO_4_ + Vydate^®^ C-LV + Max-In^®^ Sulfur – PHS	57	10.29	191 ab	22
10	ST + 28-0-0-5 + Vydate^®^ C-LV + Max-In^®^ Sulfur – PHS	52	8.47	408 ab	29
11	ST + (NH_4_)_2_SO_4_ + Vydate^®^ C-LV + Max-In^®^ Sulfur – PHS + FB	54	11.12	133 b	23
12	ST + 28-0-0-5 + Vydate^®^ C-LV + Max-In^®^ Sulfur – PHS + FB	59	12.29	464 ab	49

Notes:

z LS-means followed by the same letter are not significantly different at P ≤ 0.10 as determined by the Tukey Kramer method.

y All DP 1646 B2XF seeds were pretreated with a company fungicide and insecticide metalaxyl, pyraclostrobin, myclobutanil, imidacloprid, and fluxapyroxad.

x PHS refers to the pinhead square plant growth stage when the first additional combination of fertilizers and nematicides were applied.

w FB refers to the first bloom plant growth stage when the second additional combination of fertilizers and nematicides were applied.

v ST referees to nematicide seed treatment, Aeris^®^ (Thiodicarb) applied in 2019 and COPeO™ Prime(Fluopyram) applied in 2020.

### *Rotylenchulus reniformis* field evaluation

Plant stand was not affected by the additional fertilizer or Vydate^®^ C-LV combinations (Table 4). The additional fertilizer and nematicide combinations did not significantly increase root fresh weight at PHS + FB. *Rotylenchulus reniformis* eggs per gram of root were not significantly decreased when sampled at PHS + FB. The combination of a nematicide ST + (NH_4_)_2_SO_4_ + Vydate^®^ C-LV + Max-In^®^ Sulfur at PHS and FB had the lowest *R. reniformis* eggs per gram of root when sampled at PHS + FB. The combination of a nematicide ST + (NH_4_)_2_SO_4_ + Vydate^®^ C-LV at PHS supported a significantly larger lint yield than the treatments with no nematicide ST, which applied (NH_4_)_2_SO_4_ at PHS, 28-0-0-5 at PHS and the combination of a nematicide ST + 28-0-0-5 at PHS and FB (P ≤ 0.10). Lint yield was increased by 546 kg/ha, 603 kg/ha, 580 kg/ha or 31%, 34% and 33% with the combination of a nematicide ST + (NH_4_)_2_SO_4_ + Vydate^®^ C-LV at PHS when compared to the three lowest yielding combinations, (NH_4_)_2_SO_4_ at PHS, 28-0-0-5 at PHS and a nematicide ST + 28-0-0-5 at PHS and FB.

**Table 4:** Field trial LS means^z^ from 2019 and 2020 of the effect of nematicide and fertilizer combinations on DP 1646 B2XF^y^ stand, cotton root fresh weight at pinhead square (PHS) and first bloom (FB), *Rotylenchulus reniformis* eggs per gram of root at PHS and FB sample data summed, and yield at the Tennessee Valley Research and Extension Center.

**No**	**Treatments^y^**	**Stand^x^**	**Root fresh weigh (g) PHS^w^ + FB^v^**	***R. reniformis* eggs/g root PHS + FB**	**Lint (kg/ha)**	
1	(NH_4_)_2_SO_4_ – PHS	59	14.78	5723	1257	b^z^
2	28-0-0-5 – PHS	56	14.26	6821	1200	b
3	ST^u^ + (NH_4_)_2_SO_4_–PHS	57	16.11	4735	1345	ab
4	ST + 28-0-0-5 – PHS	56	15.99	4354	1368	ab
5	ST + (NH_4_)_2_SO_4_–PHS + FB	56	15.63	3126	1566	ab
6	ST + 28-0-0-5 – PHS + FB	55	13.51	5140	1223	b
7	ST + (NH_4_)_2_SO_4_ + Vydate^®^ C-LV – PHS	57	17.02	2558	1803	a
8	ST + 28-0-0-5 + Vydate^®^ C-LV – PHS	57	16.51	5393	1411	ab
9	ST + (NH_4_)_2_SO_4_ + Vydate^®^ C-LV + Max-In^®^ Sulfur – PHS	58	16.91	5519	1542	ab
10	ST + 28-0-0-5 + Vydate^®^ C-LV + Max-In^®^ Sulfur – PHS	55	14.89	4156	1412	ab
11	ST + (NH_4_)_2_SO_4_ + Vydate^®^ C-LV + Max-In^®^ Sulfur – PHS + FB	58	16.02	2228	1432	ab
12	ST + 28-0-0-5 + Vydate^®^ C-LV + Max-In^®^ Sulfur – PHS + FB	59	14.97	5670	1455	ab

Notes:

z LS-means followed by the same letter are not significantly different at *P* ≤ 0.10 as determined by the Tukey Kramer method.

y All DP 1646 B2XF seeds were pretreated with a BASF fungicide and insecticide metalaxyl, pyraclostrobin, myclobutanil, imidacloprid, and fluxapyroxad.

x Stand count is the number of seedlings per 7.6 m of row collected 14 DAP.

w PHS refers to the pinhead square plant growth stage when the first additional combination of fertilizers and nematicides were applied.

v FB refers to the first bloom plant growth stage when the second additional combination of fertilizers and nematicides were applied.

u ST refers to nematicide seed treatment, Aeris^®^ (Thiodicarb) applied in 2019 and COPeO^™^ Prime(Fluopyram) applied in 2020.

Mean profits for fertilizer applications of (NH_4_)_2_SO_4_ and 28-0-0-5 at PHS with no nematicides averaged $853.62 (Table 5). A nematicide ST with an additional fertilizer application at PHS increased mean profit by $46/ha from an additional fertilizer alone. A nematicide ST with an additional application of fertilizer at PHS and FB increased profit by $32/ha from an additional fertilizer alone. Combinations with Vydate^®^ C-LV applied at PHS had an increased profit of $137/ha from an additional fertilizer alone. Fertilizer and nematicide combinations that included Vydate^®^ C-LV at PHS and FB had an increased profit of $48/ha. All combinations were compared to the mean profit of treatments of an additional fertilizer with no nematicides to determine increased profit per hectare. The combination of a nematicide ST + (NH_4_)_2_SO_4_ + Vydate^®^ C-LV at PHS had the greatest overall mean profit of $1175.87 which was significantly greater than the mean profit of (NH_4_)_2_SO_4_ at PHS, 28-0-0-5 at PHS and a nematicide ST + 28-0-0-5 at PHS and FB.

**Table 5:** Field trial LS means^z^ from 2019 and 2020 representing profit mean ($/ha), and lower and upper profit determined by ANOVA (P ≤ 0.10) for fertilizer and nematicide combinations on DP 1646 B2XF^y^ at the Tennessee Valley Research and Extension Center.

**No**	**Treatments**	**Mean profit**		**Lower profit**	**Upper profit**	**Additional fertilizer and nematicide input cost^x^**
1	(NH_4_)_2_SO_4_ – PHS^w^	$862.69	b^z^	$677.97	$1047.41	$56.06
2	28-0-0-5 – PHS	$844.55	b	$659.83	$1029.27	$30.20
3	ST^v^ + (NH_4_)_2_SO_4_ – PHS	$880.33	ab	$695.61	$1065.05	$100.13
4	ST + 28-0-0-5 – PHS	$920.39	ab	$735.67	$1105.11	$77.27
5	ST + (NH_4_)_2_SO_4_ – PHS + FB^u^	$988.46	ab	$803.74	$1173.18	$153.19
6	ST + 28-0-0-5 – PHS + FB	$784.57	b	$599.84	$962.29	$107.47
7	ST + (NH_4_)_2_SO_4_ + Vydate^®^ C-LV-PHS	$1175.87	a	$991.15	$1360.59	$138.73
8	ST + 28-0-0-5 + Vydate^®^ C-LV-PHS	$913.44	ab	$728.72	$1098.16	$115.87
9	ST + (NH_4_)_2_SO_4_ + Vydate^®^ C-LV + Max-In^®^ Sulfur – PHS	$974.36	ab	$789.64	$1159.08	$150.00
10	ST + 28-0-0-5 + Vydate^®^ C-LV + Max-In^®^ Sulfur – PHS	$902.49	ab	$717.77	$1087.21	$127.14
11	ST + (NH_4_)_2_SO_4_ + Vydate^®^ C-LV + Max-In^®^ Sulfur – PHS + FB	$882.36	ab	$697.64	$1067.08	$252.93
12	ST + 28-0-0-5 + Vydate^®^ C-LV + Max-In^®^ Sulfur – PHS + FB	$922.73	ab	$738.01	$1107.45	$207.21

Notes: Revenue was calculated using prices determine by the United States Department of Agriculture upland cotton announcement of $1.32/ha in 2019 and $1.54/ha in 2020. Profit was calculated by subtracting additional input costs ($/ha) from revenue.

z LS-means followed by the same letter are not significantly different at *P* ≤ 0.10 as determined by ANOVA. Lower and upper profits are the 90% confidence levels.

y All DP 1646 B2XF seeds were pretreated with a BASF fungicide and insecticide metalaxyl, pyraclostrobin, myclobutanil, imidacloprid, and fluxapyroxad.

x Additional input costs from 2019 and 2020 were averaged to determine a single input cost for treatment analysis.

w PHS refers to the pinhead square plant growth stage, when the first combination of additional fertilizers and nematicides were applied.

v ST refers to nematicide seed treatment, Aeris^®^ (Thiodicarb) applied in 2019 and COPeO^™^ Prime (Fluopyram) applied in 2020.

u FB refers to the first bloom plant growth stage, when the second combination of fertilizers and nematicides were applied.

### *Meloidogyne incognita* field evaluation

Plant stand was not affected by the additional fertilizer or Vydate^®^ C-LV combinations (Table 6). The additional fertilizer and nematicide combinations did not significantly increase root fresh weight in samples taken at PHS + FB. *Meloidogyne incognita* eggs per gram of root were not significantly reduced in samples taken at PHS + FB. A nematicide ST + (NH_4_)_2_SO_4_ at PHS and FB had the lowest *M. incognita* eggs per gram of root, represented by the combined sample. The combination of a nematicide ST + 28-0-0-5 + Vydate^®^ C-LV + Max-In^®^ at PHS and FB had a significantly higher lint yield when compared to application of (NH_4_)_2_SO_4_ at PHS or 28-0-0-5 at PHS (*P* ≤ 0.10). Lint yield was increased by 433 kg/ha and 447 kg/ha or 35% and 38% with the combination of a nematicide ST + 28-0-0-5 + Vydate^®^ C-LV + Max-In^®^ at PHS and FB when compared to the lowest yielding treatments, (NH_4_)_2_SO_4_ at PHS and 28-0-0-5 at PHS.

**Table 6:** Field trial LS means^z^ from 2019 and 2020 of the effect of nematicide and fertilizer combinations on DP 1646 B2XF^y^ cotton root fresh weight at pinhead square (PHS) and first bloom (FB), *Meloidogyne incognita* eggs per gram of root at PHS and FB sample data summed, and yield at the Plant Breeding Unit.

**No**	**Treatments**	**Stand^x^**	**Root fresh weight (g) PHS^w^ + FB^v^**	***M. incognita* eggs/g root PHS + FB**	**Lint (kg/ha)**	
1	(NH_4_)_2_SO_4_ – PHS	44	22.36	696	832	b^z^
2	28-0-0-5 – PHS	41	22.22	1661	788	b
3	ST^u^ + (NH_4_)_2_SO_4_ – PHS	46	24.34	776	918	ab
4	ST + 28-0-0-5 – PHS	52	24.20	824	978	ab
5	ST + (NH_4_)_2_SO_4_ – PHS + FB	51	22.34	590	1044	ab
6	ST + 28-0-0-5 – PHS + FB	53	21.68	1206	1101	ab
7	ST + (NH_4_)_2_SO_4_ + Vydate^®^ C-LV – PHS	52	21.83	872	1050	ab
8	ST + 28-0-0-5 + Vydate^®^ C-LV – PHS	54	22.10	1104	1096	ab
9	ST + (NH_4_)_2_SO_4_ + Vydate^®^ C-LV + Max-In^®^ Sulfur – PHS	55	22.37	710	1051	ab
10	ST + 28-0-0-5 + Vydate^®^ C-LV + Max-In^®^ Sulfur – PHS	56	20.47	565	1052	ab
11	ST + (NH_4_)_2_SO_4_ + Vydate^®^ C-LV + Max-In^®^ Sulfur – PHS + FB	48	23.13	1093	1010	ab
12	ST + 28-0-0-5 + Vydate^®^ C-LV + Max-In^®^ Sulfur – PHS + FB	57	23.05	1215	1265	a

Notes:

z LS-means followed by the same letter are not significantly different at *P* ≤ 0.10 as determined by the Tukey Kramer method.

y All DP 1646 B2XF seeds were pretreated with a BASF fungicide and insecticide metalaxyl, pyraclostrobin, myclobutanil, imidacloprid, and fluxapyroxad.

x Stand count is the number of seedlings per 7.6 m of row collected 14 DAP.

w PHS refers to the pinhead square plant growth stage at 49 DAP, when the first combination of fertilizers and nematicides were applied.

v FB refers to the first bloom plant growth stage at 85 DAP, when the second combination of fertilizers and nematicides were applied.

u ST refers to nematicide seed treatment, Aeris^®^ (Thiodicarb) applied in 2019 and COPeO™ Prime(Fluopyram) applied in 2020.

Mean profits for fertilizer applications of (NH_4_)_2_SO_4_ and 28-0-0-5 at PHS with no nematicides averaged $548.53 (Table 7). A nematicide ST with an additional fertilizer application at PHS increased mean profit by $54/ha from an additional fertilizer application alone. A nematicide ST with an additional application of fertilizer at PHS and FB increased profit by $103/ha from an additional fertilizer application alone. Fertilizer combinations with Vydate^®^ C-LV applied at PHS had an increased profit of $93/ha from an additional fertilizer application alone. Fertilizer and nematicide combinations that included Vydate^®^ C-LV at PHS and FB had an increased profit of $131/ha. All combinations were compared to the mean profit of treatments of an additional fertilizer with no nematicides to determine increased profit per hectare. The combination of a nematicide ST + 28-0-0-5 + Vydate^®^ C-LV + Max-In^®^ Sulfur at PHS and FB had the greatest overall mean profit of $784.00 which was significantly greater than the mean profit of treatments of (NH_4_)_2_SO_4_ or 28-0-0-5 at PHS.

**Table 7:** Field trial LS means^z^ from 2019 and 2020 representing profit mean ($/ha), and lower and upper profit determined by ANOVA (P ≤ 0.10) for fertilizer and nematicide combinations on DP 1646 B2XF^y^ at the Plant Breeding Unit.

**No**	**Treatments**	**Mean profit**		**Lower profit**	**Upper profit**	**Additional fertilizer and nematicide input cost^x^**
1	(NH_4_)_2_SO_4_ – PHS^w^	$553.01	b^z^	$461.43	$644.59	$56.06
2	28-0-0-5 – PHS	$544.05	b	$452.47	$635.63	$30.20
3	ST^v^ + (NH_4_)_2_SO_4_–PHS	$569.21	ab	$477.63	$660.79	$100.13
4	ST + 28-0-0-5 – PHS	$635.85	ab	$544.27	$727.43	$77.27
5	ST + (NH_4_)_2_SO_4_–PHS + FB^u^	$608.01	ab	$516.43	$699.59	$153.19
6	ST + 28-0-0-5 – PHS + FB	$695.76	ab	$604.18	$787.34	$107.47
7	ST + (NH_4_)_2_SO_4_ + Vydate^®^ C-LV – PHS	$627.25	ab	$535.67	$718.83	$138.73
8	ST + 28-0-0-5 + Vydate^®^ C-LV – PHS	$683.50	ab	$591.92	$775.08	$115.87
9	ST + (NH_4_)_2_SO_4_ + Vydate^®^ C-LV + Max-In^®^ Sulfur – PHS	$616.22	ab	$524.64	$707.80	$150.00
10	ST + 28-0-0-5 + Vydate^®^ C-LV + Max-In^®^ Sulfur – PHS	$640.59	ab	$549.01	$732.17	$127.14
11	ST + (NH_4_)_2_SO_4_ + Vydate^®^ C-LV + Max-In^®^ Sulfur – PHS + FB	$575.53	ab	$483.95	$667.11	$252.93
12	ST + 28-0-0-5 + Vydate^®^ C-LV + Max-In^®^ Sulfur – PHS + FB	$784.00	a	$692.42	$875.58	$207.21

Notes: Revenue was calculated using prices determine by the United States Department of Agriculture upland cotton announcement of $1.32/ha in 2019 and $1.54/ha in 2020. Profit was calculated by subtracting additional input costs ($/ha) from revenue.

z LS-means followed by the same letter are not significantly different at *P* ≤ 0.10 as determined by ANOVA. Lower and upper profits are the 90% confidence levels.

y All DP 1646 B2XF seeds were pretreated with a BASF fungicide and insecticide metalaxyl, pyraclostrobin, myclobutanil, imidacloprid, and fluxapyroxad.

x Additional input costs from 2019 and 2020 were averaged to determine a single input cost for treatment analysis.

w PHS refers to the pinhead square plant growth stage, when the first additional combination of fertilizers and nematicides were applied.

v ST refers to nematicide seed treatment, Aeris^®^ (Thiodicarb) applied in 2019 and COPeO^™^ Prime (Fluopyram) applied in 2020.

u FB refers to the first bloom plant growth stage, when the second combination of additional fertilizers and nematicides were applied.

## Discussion

### *Rotylenchulus reniformis* microplot evaluation

A nematicide ST + 28-0-0-5 + Vydate^®^ C-LV + Max-In^®^ Sulfur at PHS was the most effective combination in reducing *R. reniformis* eggs per gram of root sampled at PHS + FB. The combination of a nematicide ST + (NH_4_)_2_SO_4_ + Vydate^®^ C-LV + Max-In^®^ Sulfur at PHS and FB was the most effective treatment in increasing seed cotton yield. The combination of a nematicide ST + (NH_4_)_2_SO_4_ + Vydate^®^ C-LV + Max-In^®^ Sulfur at PHS and FB increased seed cotton yields by 50% when compared to the combination of a nematicide ST + (NH_4_)_2_SO_4_ at PHS. Research supports the use of Vydate^®^ C-LV on cotton during PHS and sequential treatments reducing *R. reniformis* population levels (Hammes et al., 1999). Applications of nematicides when analyzed individually increased lint yields by 8% with a nematicide ST, 19% with a single Vydate^®^ C-LV application and 29% with a double application of Vydate^®^ C-LV.

### *Meloidogyne incognita* microplot evaluation

The combination of a nematicide ST + 28-0-0-5 + Vydate^®^ C-LV + Max-In^®^ Sulfur at PHS and FB was the most effective treatment in increasing root fresh weight sampled at PHS + FB. The application of 28-0-0-5 at PHS had the lowest *M. incognita* eggs per gram of root sampled at PHS + FB, followed by a nematicide ST + (NH_4_)_2_SO_4_ + Vydate^®^ + Max-In^®^ Sulfur at PHS and FB. Studies found that plants which had increased nitrogen available had lower nematode population levels (Miller and Wihrheim, 1966; Rodriguez-Kabana, 1986). Seed cotton yield was greatest with the combination of a nematicide ST + 28-0-0-5 + Vydate^®^ C-LV + Max-In^®^ Sulfur at PHS and FB. The combination of a nematicide ST + 28-0-0-5 + Vydate^®^ C-LV + Max-In^®^ Sulfur at PHS and FB also supported the largest root fresh weight among treatments. Similarly, a study conducted by Bednarz et al. (2000) found that cotton yields were greatest using 28-0-0-5 in a loamy sand soil type. Analyzing nematicide applications individually indicated overall seed cotton yield was increased by 7% with a nematicide ST and 6% with two foliar applications of Vydate^®^ C-LV.

### *Rotylenchulus reniformis* field evaluation

At TVREC, the combination of a nematicide ST + (NH_4_)_2_SO_4_ + Vydate^®^ C-LV + Max-In^®^ Sulfur at PHS was the most effective at increasing root fresh weight from sample data combined at PHS + FB. A nematicide ST + (NH_4_)_2_SO_4_ + Vydate^®^ C-LV + Max-In^®^ Sulfur at PHS and FB had the lowest *R. reniformis* eggs per gram of root, closely followed by the combination of a nematicide ST + (NH_4_)_2_SO_4_ + Vydate^®^ C-LV at PHS. These findings are supported by Badra and Elgindi (1979), where foliar applications of Vydate^®^ C-LV significantly reduced *R. reniformis* population levels. Similarly, the use of a nematicide ST COPeO^™^ Prime (Fluopyram) inhibited *R. reniformis* from increasing on cotton root systems (Faske and Hurd, 2015). The combination that supported the largest lint yield was a nematicide ST + (NH_4_)_2_SO_4_ + Vydate^®^ C-LV at PHS. Nematicide applications were analyzed individually in response to lint yield. An application of a nematicide ST increased overall yield by 11%. A trial conducted by Groover et al. (2020) supports this conclusion and found that a nematicide ST (COPeO^™^ Prime) increased lint yield by 14%. A single foliar application of Vydate^®^ C-LV increased overall lint yield by 13%, while two applications of Vydate^®^ C-LV increased overall lint yield by 2%. Increases in yield with single or multiple Vydate^®^ C-LV applications on cotton were also found in a study conducted by Hammes et al. (1999).

The combination with the largest mean profit in dollars/ha was a nematicide ST + (NH_4_)_2_SO_4_ + Vydate^®^ C-LV at PHS. This combination also supported the largest lint yield. Based on the lower and upper profits, there is a 90% chance when using this fertilizer and nematicide combination in a *R. reniformis* infested field, the mean profit will fall between $991.15/ha and $1360.59/ha. The increased mean profit of this combination could be contingent on the moderate fertilizer and nematicide input costs. The overall input cost of this combination was $55.96/ha which was $46.17 cheaper than the most expensive (NH_4_)_2_SO_4_ based combination. This evaluation is supported by Zimet et al. (2002) who reported financial returns in *R. reniformis* fields with lower chemical rates equating to reduced chemical input costs. Similarly, a study conducted by Koenning et al. (2007) saw an increase in yield with the use of nematicides in *R. reniformis* infested fields but when conducting an economic analysis determined the profit from the additional yield did not cover the increased chemical costs.

### *Meloidogyne incognita* field evaluation

At PBU, the combination that supported the largest root fresh weight sampled at PHS + FB was a nematicide ST + (NH_4_)_2_SO_4_ at PHS. *Meloidogyne incognita* eggs per gram of root combined were lowest in the treatment combination of a nematicide ST + (NH_4_)_2_SO_4_ + Vydate^®^ C-LV + Max-In^®^ Sulfur at PHS. This combination decreased *M. incognita* eggs per gram of root by 15% when compared to the treatment of (NH_4_)_2_SO_4_ at PHS, with no nematicide. A study conducted by Faske and Hurd (2015) also found that a nematicide ST reduced *M. incognita* population levels when compared to treatments with only a base fungicide. The combination of a nematicide ST + 28-0-0-5 + Vydate^®^ C-LV + Max-In^®^ Sulfur at PHS and FB was the most effective at increasing lint yield. This combination also supported the largest seed cotton yield in the *M. incognita* microplot trials in 2019 and 2020. The application of a nematicide ST (COPeO^™^ Prime) provided the greatest yield protection against *M. incognita* in trials conducted by Faske et al. (2021). This finding contrasts with Anderson et al. (2012) who found applications of Vydate^®^ C-LV did not have an impact on cotton lint yield. Nematicide applications were analyzed individually to evaluate lint yield responses. The application of a nematicide ST increased overall lint by 20%, a single Vydate^®^ C-LV application increased overall lint by 11%, and sequential Vydate^®^ C-LV applications increased overall lint by 5%.

The largest mean profit was obtained with the combination of a nematicide ST + 28-0-0-5 + Vydate^®^ C-LV + Max-In^®^ Sulfur at PHS and FB. This combination also supported the largest lint yields, correlating increased lint yields with additional profit. Established on lower and upper profit, there is a 90% chance that if this combination is used in a *M. incognita* populated field, profit will fall between $692.42/ha and $875.58/ha. The input cost of this combination was $83.67/ha, making it one of the most expensive treatment combinations. A study conducted by Zimet et al. (2004) found that nematicide treatments in *M. incognita* fields with fewer input costs had reduced lint yields resulting in partial net returns.

There was a decrease in *R. reniformis* eggs per gram of root and an increase in seed cotton and lint yield with the combination of a nematicide and fertilizer at either PHS or PHS + FB. Greater profit per hectare was obtained in *R. reniformis* infested soil with combinations that included at least one nematicide in combination with an additional fertilizer at either PHS or PHS + FB. The three highest profiting treatments from field trials had an additional application of (NH_4_)_2_SO_4_ at PHS or PHS + FB. The same trend was seen with *M. incognita*; eggs per gram of root were reduced and seed cotton and lint yield increased in combinations with a nematicide ST + an additional fertilizer combination at PHS or PHS + FB. Profit increased in combinations with a nematicide in combination with an additional application of fertilizer at PHS or PHS + FB. The three highest profiting treatments from field trials had an additional application of 28-0-0-5 at PHS or PHS + FB.

Our finding suggests that utilizing a nematicide with a fertilizer will increase yield and profit for growers with *R. reniformis* or *M. incognita* infested cotton fields. In *R. reniformis* field trials, 10 out of 10 fertilizer and nematicide combinations increased lint yields and 9 out of 10 combinations increased profits when compared to treatments with no nematicides. The three highest yielding combinations, a nematicide ST + (NH_4_)_2_SO_4_ at PHS and FB, a nematicide ST + (NH_4_)_2_SO_4_ + Vydate^®^ C-LV at PHS and a nematicide ST + (NH_4_)_2_SO_4_ + Vydate^®^ C-LV + Max-In^®^ Sulfur at PHS also had the largest profits. Combinations with applications of (NH_4_)_2_SO_4_ were the most economical in *R. reniformis* infested fields. These field trials suggested that the application of a nematicide ST increased lint yield by 8% and a single application of Vydate^®^ C-LV increased lint yield by 19%. The addition of a nematicide ST increased profit by $32/ha and an additional application of Vydate^®^ C-LV at PHS increased profit by $137/ha when compared to an additional fertilizer alone. In *M. incognita* field trials, 10 out of 10 fertilizer nematicide combinations increased lint yields and profit when compared to treatments with no nematicides. The three highest yielding combinations were a nematicide ST + Vydate^®^ C-LV + Max-In^®^ Sulfur at PHS and FB, a nematicide ST + 28-0-0-5 at PHS and FB, and a nematicide ST + 28-0-0-5 + Vydate^®^ C-LV at PHS also had the largest profits. Combinations with applications of 28-0-0-5 were the most economical in *M. incognita* infested fields. These trials suggested that the application of a nematicide ST increased lint yield by 20%, a single application of Vydate^®^ C-LV increased lint yield by 11% and sequential Vydate^®^ C-LV applications increased overall lint by 5%. The addition of a nematicide ST increased profit by $54/ha when compared to an additional fertilizer alone. The addition of a single application of Vydate^®^ C-LV increased profit by $93/ha and sequential applications of Vydate^®^ C-LV at PHS and FB increased profit by $131/ha. In conclusion, our hypothesis that combining nematicides with fertilizers at PHS and FB plant growth stages can provide a management system for *R. reniformis* or *M. incognita* infested cotton fields with potential for economic gains.
